# Greetings from The International Association of Environmental Mutagenesis and Genomics Societies

**DOI:** 10.1186/s41021-015-0006-8

**Published:** 2015-06-16

**Authors:** Takehiko Nohmi

**Affiliations:** Biological Safety Research Center, National Institute of Health Sciences, 1-18-1 Kamiyoga, Setagaya-ku, Tokyo 158-8501 Japan

**Keywords:** Environmental mutagenesis, Genomics, Open access, AEMGS

## Abstract

The International Association of Environmental Mutagenesis and Genomics Societies (IAEMGS) is an organization that promotes basic and applied research on environmental mutagenesis and genomics. In this article, as President of IAEMGS, I stress the important role of *Genes and Environment* to spread the voice of Asia to the international scientific community. Open access will support the journal in achieving this mission.

It is my great pleasure that the Japanese Environmental Mutagen Society (JEMS) has decided to make the society journal *Genes and Environment* open access. This journal moved to publishing only in English in 2006 and since then the society has regularly published four volumes per year and successfully spread their voice to the international scientific community. In particular, special issues in *Genes and Environment* are helpful and convenient sources to highlight important topics in environmental mutagenesis and genotoxicology, such as nanomaterials, air pollution, radiation risk, genotoxic thresholds and epigenetics.

In 2013, I was elected President of the International Association of Environmental Mutagenesis and Genomics Societies (IAEMGS). This is a global organization composed of 13 regional and national environmental mutagen societies (EMS), and the total membership is approximately 3,000. IAEMGS was established in 1973 during the 1st International Conference on Environmental Mutagens (ICEM) in Asilomar, CA, USA, to promote basic and applied research related to environmental mutagens. The original name was the International Association of Environmental Mutagens (IAEMS), and it was changed to the current name in 2013 to better reflect the interests of the member societies. IAEMGS helps to sponsor a variety of international and regional meetings including ICEM. The last ICEM (11th ICEM) was held in Iguazu, Brazil, in 2013 and the next one (12th ICEM) is planned to be held in Seoul, Korea, in 2017. International Workshops on Genotoxicity Testing (IWGT) were held as a satellite of ICEM. Other meetings supported by IAEMGS include Alexander Hollaender Courses and ICEM human populations (ICEMHP), which promote research on environmental mutagens mainly in developing countries, and the International Conference on Mechanisms of Antimutagenesis and Anticarcinogenesis (ICMAA). Therefore, it is clear that IAEMGS plays a central role in promotion of international activities of environmental mutagenesis and genomics.

IAEMGS are particularly committed to growing the presence of Asian EMS. In 2006, six Asian EMS decided to organize the Asian Association of Environmental Mutagen Societies (AAEMS). The member societies are JEMS, Korean EMS, Chinese EMS, Philippine EMS, Thailand EMS and EMS in India. To date, regional meetings, i.e., the Asian Conference on Environmental Mutagens (ACEM), have been held four times. The 1st ACEM was held in 2007 in Kitakyushu, Japan; the 2nd ACEM was held in 2010 in Pattaya in Thailand; the 3rd ACEM in 2012 in Hangzhou in China; and the 4th ACEM in 2014 in Kolkata, India. In 2010, the Iranian EMS has joined AAEMS. Thus, seven EMS constitutes AAEMS. The total membership is more than 2,000, which accounts for 2/3 of the membership of IAEMGS. The elevated activities in Asian countries may reflect the fact that economies and industries are moving upward in Asia, which induces environmental problems such as air pollution and food safety issues in the region. However, local environmental problems are sometimes missed or overlooked by people in other nations or regions even though the problem is quite severe in a local area. For example, I myself learned that arsenic in drinking water was a severe social environmental problem in Western Bengal in India and Bangladesh when I first visited Kolkata, India, in 2008 in the 14th Alexander Hollaender Course on Genetic Toxicology. Before this visit, I had no idea how seriously arsenic affects the local people’s health. Therefore, I expect that the journal *Genes and Environment* would spread not only the Japanese domestic voice but also the Asian, and international, voice to the global research community. In this respect, open access is the most appropriate model for this journal.

The name *Genes and Environment* was given because most human diseases, such as cancer, involve interactions between genes and the environment. Thanks to advanced DNA-sequencing technology, very detailed genomic information is available for studying cancer cells. However, affecting environmental factors including endogenous ones, such as inflammation, are still poorly characterized at the molecular level. In this respect, I do hope that *Genes and Environment* will contribute to elucidating the environmental factors affecting human diseases. Through this work, we could approach solving the complex problem of interactions between genes and environment in human diseases.

## Abbreviations

JEMS: The Japanese Environmental Mutagen Society
IAEMGS: The International Association of Environmental Mutagenesis and Genomics Societies
ICEM: International Conference on Environmental Mutagens
IAEMS: The International Association of Environmental Mutagens
IWGT: International Workshops on Genotoxicity Testing
ICEMHP: ICEM human populations
ICMAA: The International Conference on Mechanisms of Antimutagenesis and Anticarcinogenesis
AAEMS: The Asian Association of Environmental Mutagen Societies
ACEM: The Asian Conference on Environmental Mutagens

